# Reactive Oxygen Species: Molecular Mechanisms, Cellular Targets, and Implications for Genomic Stability

**DOI:** 10.1155/bmri/6918118

**Published:** 2026-07-31

**Authors:** Alkhaddour Aziz, Zam Wissam

**Affiliations:** ^1^ Lab of Molecular Neurobiology, Institute of Physical and Organic Chemistry, Southern Federal University, Rostov-on-Don, Russia, sfedu.ru; ^2^ Department of Analytical and Food Chemistry, Faculty of Pharmacy, Tartous University, Tartous, Syria, tartous-univ.edu.sy

**Keywords:** aging, apoptosis, mitochondrial dysfunction, oxidative lesions, oxidative stress, ROS

## Abstract

Reactive oxygen species (ROS) are highly reactive molecules generated through endogenous metabolic pathways and exogenous environmental exposures. While essential for physiological processes—including cell signaling, proliferation, differentiation, immune defense, and neurotransmission—dysregulated ROS production contributes to oxidative stress and widespread biomolecular damage. This review outlines the major enzymatic and non‐enzymatic mechanisms of ROS formation, emphasizing mitochondrial electron leakage, NADPH oxidase activity, and metal‐catalyzed reactions. It further explores the impact of cold exposure, physical exercise, nutritional imbalance, and aging on ROS levels through alterations in mitochondrial function, calcium signaling, and antioxidant defenses. While ROS are vital for certain biological activities, the article also emphasizes their destructive potential. Particular attention is given to the vulnerability of mitochondrial DNA (mtDNA) and nuclear DNA to hydroxyl radical attack, resulting in base modifications, sugar lesions, tandem lesions, and DNA–protein cross‐links. These lesions disrupt replication fidelity, impair DNA repair, and promote mutagenesis, ultimately threatening genomic stability. Finally, apoptosis is described as being modulated by ROS in a dose‐dependent manner through the intrinsic, extrinsic, and ER–stress pathways, with the central role of p53 in determining cell fate being highlighted. Collectively, this review integrates current knowledge on ROS generation, physiological functions, stress‐induced dysregulation, and the molecular mechanisms underlying oxidative damage, offering a comprehensive perspective on their implications for genomic integrity and disease development.

## 1. Introduction

Free radicals are chemical species, molecules, or ions that possess one or more unpaired electrons, making them highly reactive. They originate from endogenous sources, such as mitochondrial respiration, cytochrome P‐450 metabolism, peroxisomal activity, and the activation of inflammatory cells [1]. They can also be produced through the effect of exogenous factors such as exposure to X‐rays and ultraviolet radiation, metal‐catalyzed reactions, environmental pollutants, and immune cell activity during inflammation [2]. Among these, reactive oxygen species (ROS) are considered key metabolic byproducts and can be classified into radicals and non‐radicals based on their chemical behavior [3, 4]. ROS are commonly used to define the reactive molecules and free radicals originating from molecular oxygen. Physiologically significant endogenous ROS include superoxide anion (O₂˙^−^), hydroxyl radical (OH˙), hydrogen peroxide (H₂O₂), hypochlorous acid (HOCl), peroxyl radical (ROO˙), and hydroperoxyl radical (HOO˙).

**Figure 1:**
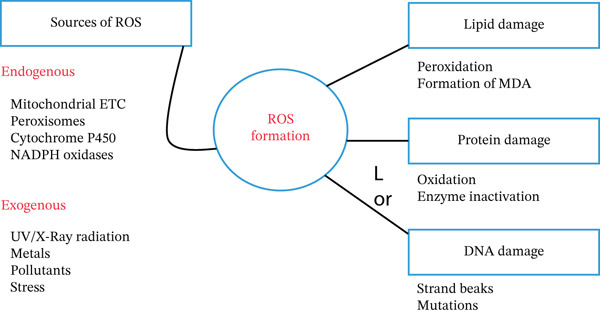
Formation and biological impact of reactive oxygen species.

Beyond their chemical reactivity, ROS play essential roles in cellular signaling, immune defense, proliferation, differentiation, and metabolic regulation. They play crucial roles in innate immunity via NADPH oxidase (NOX) activation, as well as in metabolic regulation within mitochondria [5]. However, when their production exceeds antioxidant capacity, ROS initiate oxidative stress that disrupts cellular membranes (lipid peroxidation [LPO]), impairs protein function and structural integrity, and causes genetic mutations or instability (DNA damage) as presented in Figure 1 [4]. This dual nature positions ROS as both indispensable regulators of physiology and major contributors to pathological processes.

Given the growing recognition of ROS as central mediators of genomic instability, this review examines their mechanisms of formation, their physiological and stress‐induced roles, and the molecular pathways through which they damage lipids, proteins, mitochondrial DNA, and nuclear DNA. Special emphasis is placed on complex oxidative lesions—including base modifications, sugar damage, tandem lesions, and DNA–protein cross‐links (DPCs)—and their implications for mutagenesis, aging, and apoptosis.

## 2. Mechanism of Generation of Free Radicals

Several key reactive species play crucial roles in cellular processes and oxidative stress. Superoxide anion (O₂˙^−^), containing one unpaired electron, is enzymatically produced by xanthine oxidase and cytochrome P450 in both the mitochondria and cytoplasm. It also forms through the auto‐oxidation of oxygen during the mitochondrial electron transport chain and is subsequently converted into H₂O₂ by the enzyme superoxide dismutase (SOD). H₂O₂, which carries two electrons, is less reactive but can be reduced to water by glutathione peroxidase (GPX) in the mitochondria and cytoplasm, or by catalase in peroxisomes [6]. Among all free radicals, the hydroxyl radical (˙OH)—with three unpaired electrons—is the most reactive. It is generated either through the radiolysis of water or via the Fenton reaction, where H₂O₂ reacts with ferrous ions (Fe^2+^). Nitric oxide (NO), produced by various cells such as macrophages, neurons, and endothelial cells, can react with superoxide to form peroxynitrite (ONOO^−^), a highly reactive and damaging species. Peroxyl radicals (ROO˙) are formed by a direct reaction of oxygen with alkyl radicals (R˙). Decomposition of alkyl peroxides (ROOH) also results in peroxyl radical (ROO˙) and alkoxyl (RO˙) radicals [7]. The simplest form of peroxyl radical is perhydroxyl radical (HOO˙), which is formed by the protonation of superoxide [8]. Additionally, halide reagents like chloride, released by leukocytes, can combine with superoxide to form hypochlorous acid (HOCl), a potent cytotoxic agent involved in immune defense.

## 3. ROS in Normal Physiology

ROS at low‐to‐moderate concentrations regulate crucial processes like cellular development, proliferation, differentiation, immune responses, and neurotransmission. ROS play an important role in the maintenance of self‐renewal, proliferation, and differentiation of stem cells. The production of ROS in stem cells is tightly regulated to ensure that they have the ability to maintain tissue homeostasis and repair damaged tissues for the life span of an organism [9]. They exert their influence by reversibly oxidizing the active sites of transcription factors such as nuclear factor‐kappa B (NF‐*κ*B) and activator protein‐1 (AP‐1), which are part of the mitogen‐activated protein kinase (MAPK) signaling pathways [10]. Studies have demonstrated that platelet‐derived growth factor (PDGF) and epidermal growth factor (EGF) can rapidly and transiently elevate ROS levels via NOXs, and this increase is crucial for growth‐factor‐induced receptor tyrosine phosphorylation [11]. Furthermore, ROS‐mediated oxidation enhances the binding of EGF to its receptors, including the epidermal growth factor receptor (EGFR) [12]. In mammals, RNA polymerase III (Pol III) is subject to redox regulation [13]. The formation of a transcriptionally active pre‐initiation complex in vertebrates is governed by the redox‐sensitive transcription factor TFIIB‐related factor 2 (Brf2), suggesting that a eukaryotic nuclear RNA polymerase can directly respond to redox changes to regulate the transcription of essential RNAs, potentially initiating apoptosis. Ultimately, cell growth and cell cycle arrest are tightly controlled through complex redox‐dependent mechanisms [14]. Increasing evidence indicates ROS could impact the wound healing process by regulating inflammatory response, cell proliferation, angiogenesis, granulation as well as extracellular matrix formation [15]. Additionally, ROS act as second messengers that regulate neurotransmission and structural changes in neurons required for learning and memory. They have been implicated as modulators of hippocampus‐dependent and hippocampus‐independent memory formation by mediating synaptic plasticity, including long‐term potentiation (LTP) and long‐term depression (LTD). They also influence other signaling molecules, including CaMKII and ERK, which are necessary for memory formation [16]. ROS also play a pivotal role in the synthesis of various biologically important compounds, including prostaglandins and thyroxine [17]. In thyroid cells, the production of thyroxine is tightly regulated by the concentration of H_2_O_2_, which is essential for catalyzing the iodination of thyroglobulin. Beyond their role in biosynthesis, ROS are integral to the immune system and numerous physiological processes.

## 4. ROS‐Mediated Regulation of Cellular Signaling

ROS modulate numerous redox‐sensitive signaling pathways, playing a central role in cellular regulation. They catalyze the oxidation of cysteine residues in tyrosine kinases and mitogen‐activated protein (MAP) kinase phosphatases—specifically protein tyrosine phosphatases—thereby reversibly inhibiting enzymatic activity and influencing growth factor‐mediated signaling pathways [18]. In mammals, ROS signaling is tightly regulated by GPX and peroxiredoxins (PRXs), which modulate H₂O₂ signaling downstream of tyrosine kinases and cytokine receptors [19]. PRX1 enhances cell survival by activating p38 MAPK, while PRX2 suppresses H₂O₂ and TNF‐*α*‐induced upregulation of NF‐*κ*B [20]. Another layer of ROS regulation involves the modulation of antioxidant systems. NO activates the tumor suppressor p53, which reduces H₂O₂ accumulation by upregulating GPX1 [21]. Additionally, NO inactivates Hdm2—a ubiquitin ligase—through S‐nitrosylation, preventing its interaction with p53 and thereby inhibiting p53 ubiquitination and degradation [19]. The oncogene c‐Myc promotes glutathione (GSH) synthesis, amplifying its oncogenic potential [22]. H₂O₂ also activates transcription factors from the forkhead box O (FOXO) family, contributing to redox‐sensitive gene regulation [19]. The NRF2‐KEAP1 pathway is a dedicated system for detoxifying oxidants and xenobiotics. Upon oxidative stress or electrophilic attack, NRF2 is released from KEAP1 due to phosphorylation and oxidative modification of KEAP1’s cysteine residues [19]. NRF2 then translocates to the nucleus, where it interacts with various proteins and antioxidants to initiate gene expression, ultimately protecting cells from oxidative damage.

According to established literature, various kinases play a central role in the cellular response to DNA damage by coordinating the detection, signal transduction, and repair of lesions, thereby safeguarding genomic integrity. Key among these are ATM (Ataxia Telangiectasia Mutated), ATR (ATM and Rad3‐related), and DNA‐PK (DNA‐dependent Protein Kinase), all of which are integral to the DNA damage response (DDR) cascade [23–26]. ATM is predominantly activated by double‐strand breaks (DSBs) and facilitates repair via homologous recombination (HR) through the phosphorylation of critical substrates, including p53 and CHK2. In contrast, ATR responds to replication stress (RS) and single‐stranded DNA, stabilizing stalled replication forks and orchestrating repair predominantly through CHK1 activation. Meanwhile, DNA‐PK is indispensable for non‐homologous end joining (NHEJ), where it mediates the processing and ligation of DNA termini at DSB sites. Additional kinases, such as CHK1 and CHK2, enforce cell cycle checkpoints to prevent progression until DNA lesions are resolved, while CDKs (Cyclin‐dependent kinases) and PLK1 (Polo‐like kinase 1) modulate cell cycle dynamics in response to damage or recovery. Furthermore, stress‐activated kinases like MAPKs influence apoptotic pathways, and Aurora kinases ensure mitotic arrest until genomic integrity is restored. Collectively, these kinases not only promote cell survival through efficient repair but also initiate apoptosis when damage proves irreparable [27–29].

Despite these robust repair mechanisms, genomic alterations—including amplifications, deletions, or mutations in tumor suppressor genes and oncogenes—can still arise, contributing significantly to cancer development^34^. DNA damage is now recognized as a hallmark of malignancy, with cancer cells exhibiting heightened genomic instability and aberrant damage profiles [30]. Mutational selection pressures acting on proto‐oncogenes, tumor suppressor genes, and genomically unstable regions further drive the malignant transformation of normal cells^37^. Notably, the upregulation of DNA repair genes and the downregulation of DNA relocation genes present promising opportunities for targeted cancer therapies, particularly through multitarget anticancer agents that simultaneously inhibit repair mechanisms and disrupt oncogenic signaling pathways [31].

The three principal DDR pathways—mediated by ATM, ATR, and DNA‐PK—serve as central sentinels of genomic stability, exerting considerable influence over tumor progression and therapeutic outcomes. These pathways govern a range of essential cellular processes, including cell cycle control, apoptosis, gene expression, oxidative stress management, and telomere maintenance. Given their extensive involvement in these critical functions, DDR pathways represent compelling therapeutic targets [32]. Nevertheless, translating these insights into clinical practice demands a more nuanced understanding to maximize therapeutic efficacy while mitigating the potential risks associated with pathway inhibition [33].

## 5. The Role of Stress in ROS Induction

**Figure 2:**
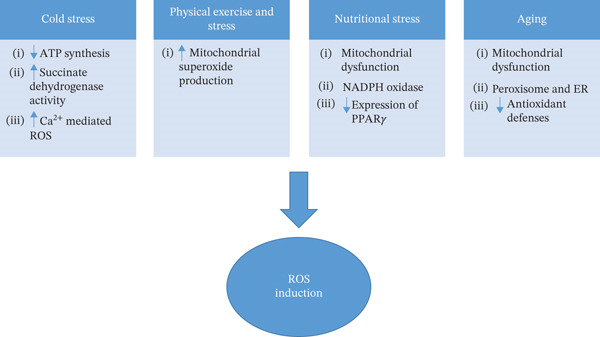
Multifaceted relationship between stress and ROS.

There is a multifaceted relationship between stress and ROS as illustrated in Figure 2. Cold stress, physical exercise, nutritional imbalance, and aging each activate distinct molecular pathways that converge on ROS production. These stressors disrupt mitochondrial function, modulate calcium signaling, and impair antioxidant defenses, ultimately contributing to oxidative stress and cellular damage.

### 5.1. Cold Stress

By definition, cold stress is a physiological condition that arises when ambient temperatures fall below 18°C, leading to adverse effects on the body’s metabolic functions. One of the key consequences of cold stress is a reduction in ATP synthesis, which triggers a shift in post‐stress perfusion patterns, activating glycolytic pathways to compensate for the reduced mitochondrial output. Notably, an increase in succinate dehydrogenase activity has been observed, indicating that cold stress directly influences the Krebs cycle and mitochondrial respiration [34]. These metabolic disruptions result in an imbalance in energy production, which in turn promotes the generation of ROS, contributing to oxidative stress and potential cellular damage.

In lung epithelial cell, researchers observed an increase of ROS and [Ca^2+^] mediated by cold stress and completely attenuated by removing Ca^2+^ from medium. Results showed that (TRPA1) agonist (allyl isothiocyanate, AITC) played an important role in the enhanced production of ROS [35].

### 5.2. Physical Exercise and Stress

The impact of exercise on ROS varies from harmful to beneficial and depends on the type of exercise [36]. Acute exercise induces ROS production and regular exercise training increases the endogenous antioxidative system and protects the body against adverse effects of oxidative damage [37]. In a study conducted by Cho et al., a single bout of exhaustive exercise increased both oxidant and antioxidant status, but the increase in antioxidant status was not enough to scavenge the high increase in oxidants leading to oxidative damage [38]. However, long‐term aerobic exercise training with moderate intensity increased SOD activity, which reduces oxidative damage [39]. Notably, mitochondrial ROS generated during regular physical activity are essential for activating the primary signaling cascades involved in muscle adaptation [40]. One of the central regulators of the antioxidant response is nuclear factor erythroid 2‐related factor 2 (Nrf2), which governs the expression of various antioxidants and cytoprotective molecules [41]. High‐intensity exercise has been associated with increased Nrf2 expression [42], and this response is further amplified by the upregulation of peroxisome proliferator‐activated receptor‐*γ* coactivator‐1*α* (PGC‐1*α*), a key driver of mitochondrial biogenesis [43]. PGC‐1*α* also enhances Nrf2 activity, creating a feedback loop that supports cellular resilience. Additionally, redox‐sensitive transcription factors such as MAPK and nuclear factor kappa B (NF‐*κ*B) are upregulated in response to oxidative signals, further contributing to the adaptive remodeling of muscle tissue [44, 45].

### 5.3. Nutritional Stress

Encompassing both overnutrition and malnutrition, triggers increased ROS. Malnutrition, including both undernutrition and protein deficiency, is characterized by mitochondrial dysfunction and oxidative stress in peripheral blood leukocytes at the level of the mitochondrial complex I. This induces high levels of ROS production, which activates inflammation pathways, and stimulates the production of adhesion molecules in endothelial cells [46]. A large number of studies within the last decade indicated that overnutrition induces ROS formation and oxidative stress. A meal high in fats, proteins, and/or carbohydrates causes both an increase in the release of insulin and an increase in the generation of reactive ROS mainly through mitochondrial overload and endoplasmic reticulum (ER) stress. The presence of oxidizable substrates during rest increases the potential of the mitochondrial membrane, which in turn reduces the rate of oxidative phosphorylation. This increases the likelihood that electrons will “leak” from the respiratory chain to molecular oxygen, which will produce reactive ROS [47]. Consequently, the diminished substrate utilization is accompanied by an increment in NADH, in both the cytosol and in mitochondria [48]. In addition, NOXs have also been described to contribute to elevated ROS levels and to be upregulated in the liver in several animal models [49]. Additionally, decreased expression of peroxisome proliferator‐activated receptors (PPAR*γ*) in morbidly obese persons was associated with an increase in the concentration of free fatty acids after a fat meal which increases their oxidation in peroxisomes and the generation of H₂O₂ [50].

### 5.4. Aging

Aging is a complex biological process defined as the gradual decline in an organism’s physiological functions [51]. This process is closely linked to the accumulation of oxidative damage caused by ROS, which randomly and cumulatively impair macromolecules such as proteins, lipids, and nucleic acids. These disruptions contribute to cellular dysfunction and ultimately cell death, aligning with the free radical theory of aging. Nevertheless, every individual has its own aging mechanisms due to differences in environmental conditions and genetics. However, oxidative stress theory that impact mitochondria, as a primary endogenous source of ROS, is considered one of the famous theories which largely contribute to the aging process [52]. During the aging process, mitochondrial dysfunction is one of the most important mechanisms contributing to decline in ATP production and elevated ROS production together with a decline in the antioxidant defense [53]. Additionally, ROS‐induced damage affects mitochondrial components including proteins, lipids, and mitochondrial DNA (mtDNA), compromising cellular energy production and integrity [54]. Moreover, besides mitochondria, other important cell organelles such as peroxisome and ER also produce ROS that contribute to aging [52]. Additionally, aging along with elevated ROS levels is associated with a decline in the antioxidant defenses mainly GPX, GSH reductase, catalase, NADPH‐quinone oxidoreductase‐1 (NQO1), heme‐oxygenase (HO‐1), thioredoxin (Trx), and sulfiredoxin (Srx) [55]. The classical free radical theory of aging has been challenged by compelling evidence from studies in yeast, worms, and flies, which consistently demonstrate that inhibiting mitochondrial respiration prolongs lifespan and delays aging. This paradoxical benefit of mitochondrial stress is encapsulated by the term mitohormesis—a fusion of mitochondria and hormesis. Mitohormesis has been defined as the hormetic response to sublethal mitochondrial stress that promotes health and vitality. A growing body of evidence now firmly establishes that low‐level mitochondrial stress confers advantages rather than harm to organismal health [56]. What factors determine whether mitochondrial stress yields beneficial or adverse consequences? First, the stress level may be critical, as indicated by the hormesis concept. Second, the spatiotemporal context of stress may influence the ability to trigger a mitohormetic response. For example, in *Caenorhabditis elegans*, mitochondrial stress during larval stages extends lifespan, whereas the same stress applied in adulthood has no such effect [57]. Thus, mitochondrial perturbations in early life may have lifelong positive impacts on health and longevity. In this regard, regular, moderate‐intensity exercise may represent an effective and safe strategy to induce mitohormetic effects across multiple tissues [58]. Additionally, mitokines such as GDF15, FGF21, and MOTS‐c, along with their modified analogues, could serve this purpose. While the favorable effects of mitochondrial stress on longevity have been largely documented in lower organisms, emerging evidence suggests that agents eliciting mild mitochondrial stress (metformin) and those inducing mitonuclear protein imbalance (resveratrol and NAD^+^ precursors such as nicotinamide riboside and nicotinamide mononucleotide) may have the potential to delay aging and extend lifespan in mammals. Therefore, a deeper understanding of mitohormesis and the development of mitohormesis‐targeting agents may open new therapeutic avenues for many human diseases associated with obesity and aging [59].

### 5.5. Mechanistic Framework for Stress‐Induced ROS Dysregulation

Environmental stresses that increase cellular energy demand—including exercise and cold exposure—trigger mitochondrial biogenesis [60, 61]. Numerous studies have demonstrated that both exercise and cold exposure upregulate peroxisome proliferator‐activated receptor gamma coactivator‐1 alpha (PGC‐1*α*) expression in skeletal muscle and adipose tissue [62, 63]. PGC‐1*α* serves as the master regulator of mitochondrial biogenesis, acting through its interaction with nuclear respiratory factor‐1 (NRF‐1), which in turn activates mitochondrial transcription factor A (Tfam) to coordinate the expression of mitochondrial proteins [64]. Exercise elevates cellular energy demand, leading to increased intracellular AMP, Ca^2+^ concentrations, free phosphate groups (Pi), and ROS [65]. These molecules function as potent signaling transducers, activating calcium/calmodulin‐dependent protein kinases (CaMK), AMP‐activated protein kinase (AMPK), and p38 mitogen‐activated kinase (p38MAPK), which subsequently trigger the transcription and activation of PGC‐1*α* and drive mitochondrial biogenesis [66]. In contrast, cold exposure is first sensed by peripheral sensory nerves; this information is relayed to the hypothalamus, which orchestrates sympathetic nervous system (SNS) activity, leading to adrenergic hormone release onto adipocytes. Adrenergic stimulation activates G protein‐coupled *β*‐adrenergic receptors, promoting cell proliferation and inducing multiple adaptive changes in adipose tissue, including increased mitochondrial content [67, 68]. However, it remains unclear whether exercise and cold exposure act independently or interdependently, as previous studies have typically examined the effects of exercise^6,8,16^ or cold exposure [62–64] in isolation. Moreover, whether these two stimuli converge on a common mechanistic pathway or operate through distinct mechanisms to achieve a shared phenotypic outcome remains an open question [69].

## 6. Damaging Reactions of Free Radicals

ROS are highly reactive molecules that attack all major classes of biomolecules, with polyunsaturated fatty acids (PUFAs) in cell membranes being especially vulnerable. The oxidative degradation of PUFAs, known as LPO, is a destructive process that contributes to the pathogenesis of chronic diseases such as cancer, coronary heart disease (CHD), and osteoporosis [70]. The mechanism of LPO involves a chain reaction initiated by an oxidizing radical (Rc), which abstracts a hydrogen atom from a PUFA (LH), forming a lipid radical (Lc). This radical rapidly reacts with molecular oxygen to produce a lipid peroxyl radical (LOOc), which propagates the reaction by attacking other PUFA molecules, generating lipid hydroperoxides (LOOH). These hydroperoxides are unstable and can decompose into additional radical species, perpetuating cellular damage and amplifying oxidative stress, as illustrated in Equations (1)–(4) and supported by reference [71].

(1) LH+Rc⟶Lc+RH

(2) Lc+O2⟶LOOc

(3) LOOc+LH⟶LOOH+Lc

(4) LOOH⟶LOc+LOOc+aldehydes

Among PUFAs, arachidonic acid and docosahexaenoic acid are especially susceptible to oxidation. This LPO process generates reactive aldehydes like 4‐hydroxynonenal (4‐HNE) and malondialdehyde (MDA), which can further propagate oxidative damage. In addition to lipids, proteins are also vulnerable; ROS can oxidize both the side chains and backbones of proteins. Although all amino acids can be modified by ROS, cysteine, and methionine sulfur groups are the most susceptible to oxidative changes. This oxidative modification often results in the formation of carbonyl groups on amino acid residues, altering several physical and chemical properties, including conformation, structure, solubility, susceptibility to proteolysis, and enzyme activities [72].

### 6.1. The Free Radicals and Mitochondrial DNA (mtDNA)

In animals, mitochondrial DNA (mtDNA) is maternally inherited, with each oocyte containing approximately 10^5^ copies, while somatic cells typically harbor around 10^3^–10^4^ copies. During replication in somatic cells, mutations arising in a single mtDNA molecule can clonally expand. As cells divide, mitotic segregation randomly distributes mtDNA mutations, potentially resulting in daughter cells that are either homoplasmic—containing only normal or mutant mtDNA—or heteroplasmic—harboring a mix of both. This stochastic distribution explains how cells with initially low levels of mutated mtDNA can give rise to progeny with high mutation loads, a phenomenon governed by mitotic segregation. These mutations may include point mutations or large‐scale deletions [73].

Superoxide, the primary ROS generated by the mitochondrial respiratory chain, cannot traverse mitochondrial membranes and must be converted to H₂O₂ by SOD. Unlike superoxide, H₂O₂ can diffuse out of mitochondria and affect the entire cell. Through Fenton chemistry, H₂O₂ reacts with transition metals like iron to produce hydroxyl radicals—the most reactive ROS, limited only by diffusion. Hydroxyl radicals can inflict severe DNA damage, including single‐strand breaks (SSBs), abasic sites, intrastrand adducts, DNA–protein crosslinks, and base modifications [74]. Within mitochondria, such damage compromises mtDNA integrity, potentially impairing complexes I and III of the electron transport chain. This dysfunction enhances superoxide production, amplifying oxidative stress by reducing the synthesis of essential electron transport proteins. The resulting feedback loop exacerbates mitochondrial damage and ROS generation, ultimately triggering apoptosis [75].

### 6.2. Mechanisms of Free Radical‐Induced Damage to DNA

**Figure 3:**
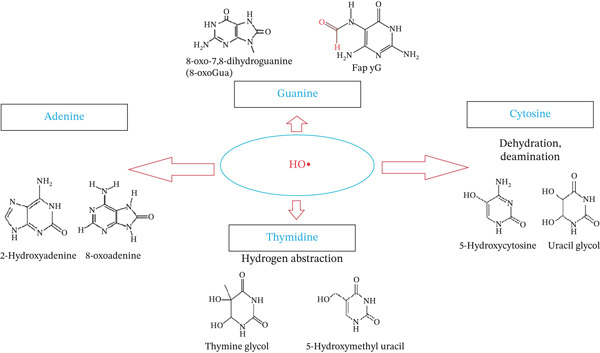
ROS‐induced oxidative damage to DNA nucleobase.

Hydroxyl radicals (HO˙), among the most reactive species of ROS, interact with DNA primarily through two mechanisms: addition to the aromatic moieties of DNA bases and hydrogen abstraction. Approximately 70% of these reactions occur on DNA bases, leading to SSBs, while the remaining 30% affect the deoxyribose backbone [76]. ROS can oxidatively damage all four DNA nucleotides (Figure 3), resulting in over 100 distinct types of base lesions, including oxidized aromatic derivatives, fragmented bases, and ring‐opened structures. These alterations disrupt normal base‐pairing, often leading to transition mutations (purine‐to‐purine or pyrimidine‐to‐pyrimidine) or transversion mutations (purine‐to‐pyrimidine or vice versa) [77].

The schematic illustrates representative oxidation pathways for adenine, guanine, cytosine, and thymidine. Carbon positions involved in hydroxylation, deamination, or hydrogen abstraction are indicated in the text, and oxidized nucleotide species (e.g., 8‐oxo‐7,8‐dihydroguanine, 8‐oxoadenine, thymine glycol, 5‐hydroxycytosine) are shown to highlight structural alterations resulting from radical attack.

**Figure 4:**
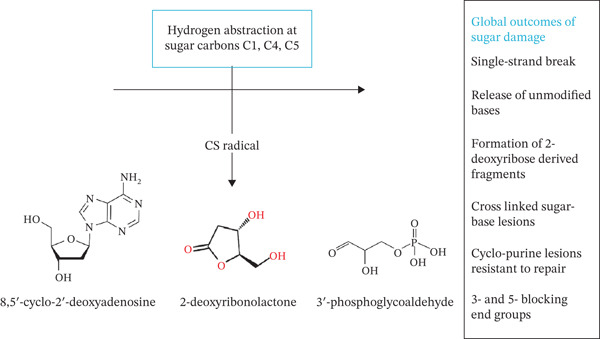
ROS induced damage pathways in the sugar moiety of DNA.

Beyond base oxidation, the sugar moiety of DNA is a major target of HO˙ attack (Figure 4). Hydrogen abstraction from the deoxyribose ring generates carbon‐centered sugar radicals at positions C1 ^′^, C3 ^′^, C4 ^′^, and C5 ^′^. These unstable intermediates undergo *β*‐elimination, oxidation, or rearrangement reactions that produce a variety of sugar‐derived lesions. Such changes in nucleobase structure significantly contribute to the mutation burden associated with numerous human diseases [77].

The schematic illustrates hydrogen abstraction at key carbon positions (C1, C4, and C5) of the deoxyribose ring, leading to formation of carbon‐centered radicals and subsequent oxidative products. Oxidized sugar derivatives, including 2‐deoxyribonolactone and 3 ^′^‐phosphoglycoaldehyde, are shown to highlight structural alterations resulting from hydroxyl radical attack.

#### 6.2.1. Guanine

Guanine, one of the four DNA nucleobases, is highly susceptible to oxidative damage by ROS, particularly hydroxyl radicals (˙OH) because the oxidation potential of guanine is lower than the other DNA bases [78]. This interaction primarily occurs through addition at the C8 position of the guanine base, forming 8‐hydroxy‐7,8‐dihydroguan‐8‐yl radicals. In the presence of oxygen, these radicals undergo one‐electron oxidation to yield 8‐oxo‐7,8‐dihydroguanine (8‐oxoGua), a well‐known mutagenic lesion. Alternatively, one‐electron reduction leads to imidazole ring opening, resulting in the formation of 2,6‐diamino‐4‐hydroxy‐5‐formamidopyrimidine (Fapy‐Gua). Both 8‐oxoGua and Fapy‐Gua are major products of guanine oxidation and contribute to genomic instability by promoting mispairing during DNA replication, often leading to G → T transversions. These lesions are central to the mutation burden associated with oxidative stress and are implicated in various human diseases [79]. Several other lesions are formed in high abundance, including 5‐carboxamido‐5‐formamido‐2‐iminohydantoin (2Ih), a prevalent product in in vitro chemistry that is challenging to study from cellular sources [80].

#### 6.2.2. Cytosine

Cytosine, one of the four DNA bases, is susceptible to oxidative modifications that can significantly alter its chemical structure and biological function. Under oxidative stress, cytosine can undergo dehydration and deamination, leading to the formation of cytosine glycol intermediates. These intermediates further degrade into distinct products, including 5‐hydroxycytosine, 5‐hydroxyuracil, and uracil glycol. Among the oxidized cytosine products, 5‐hydroxycytosine, and 5‐hydroxyuracil have been reported to be the major stable products [81]. Whereas, uracil glycol and 5‐hydroxyuracil showed the highest mutation frequencies [81]. Several recent studies have established that the oxidized cytosine product 5‐hydroxy uracil (5‐OHU) as the major chemical precursor to the GC to AT transition mutations [82].

#### 6.2.3. Adenine

Adenine interacts with hydroxyl radicals (˙OH) to form 8‐oxoadenine and, to a lesser extent, 2‐hydroxyadenine [83]. 2‐Hydroxyadenine is potentially miscoding as its replication is mutagenic and caused base substitutions. Thermodynamic analysis showed that 2‐hydroxyadenine forms stable base pairs with T, C, and G, and to a lesser extent with A, allowing it to act as a promiscuous base in DNA replication [84]. Additionally, these adducts can undergo dehydration to yield Ade(–H)˙ radicals that can first be protonated to form Ade˙+ and then regenerates C8‐OH– adduct radical upon hydration. This regenerated radical is chemically identical to the one formed by direct ˙OH addition to adenine’s C8 position. Through these pathways, adenine can ultimately be converted into 8‐hydroxyadenine (8‐OH‐Ade), a mutagenic lesion associated with oxidative DNA damage [85].

#### 6.2.4. Thymidine

Thymidine undergoes oxidative modification when a hydride anion (H˙) is abstracted from its methyl group, forming an allyl radical. This intermediate participates in further oxidation and reduction reactions, ultimately generating a variety of DNA lesions. These include 5,6‐dihydroxy‐5,6‐dihydrothymidine (thymidine glycol) and 5‐hydroxymethyl‐2 ^′^‐deoxyuridine [86]. Thymine can also suffer hydrogen abstraction from its methyl group, giving 5‐(hydroxymethyl)uracil (5‐OH‐MeUra) after coupling with a hydroxyl radical. Thymine glycol (5,6‐dihydroxy‐5,6‐dihydrothymine) is one of the principal DNA lesions as it blocks replicative DNA polymerases, potentially leading to lethal, non‐mutagenic stalling in cells [87].

#### 6.2.5. Mechanisms of Damage to the Sugar Moiety of DNA

Hydroxyl radicals (OH) are potent agents of DNA damage, particularly targeting the carbon atoms of the sugar backbone of DNA—2‐deoxyribose. The abstraction at C1 ^′^ gives 2‐deoxyribonolactone, at C5 ^′^ gives 3 ^′^‐phosphoglycoaldehyde, and at C4 ^′^ gives an intermediate unsaturated dialdehyde that can couple with cytosine to form dCyd341 [88]. The formation of dCyd341 is particularly problematic for DNA repair mechanisms because it is accompanied by a strand break, complicating the restoration of DNA integrity. In addition, the C5 ^′^‐centered radicals of 2‐deoxyribose can react with the purine ring in the same nucleoside to produce 8,5 ^′^‐cyclo‐2 ^′^‐deoxyguanosine (8,5 ^′^‐cyclo‐dGuo) or 8,5 ^′^‐cyclo‐2 ^′^‐deoxyadenosine (8,5 ^′^‐cyclo‐dAdo), which are among the major lesions in DNA that are formed by attack of hydroxyl radical [88]. The resulting damage includes strand breaks, release of unmodified DNA bases, and the formation of various 2‐deoxyribose‐derived compounds. Some of these products remain attached as end groups on broken DNA strands, while others are completely released from the DNA molecule [89].

#### 6.2.6. Mechanisms of Formation of Tandem Lesions

ROS generate a type of DNA damage called tandem lesions, two adjacent nucleotides both modified. Tandem lesions are commonly formed when a peroxyl pyrimidine radical attaches to the C8 position of a neighboring purine base. These tandem lesions include combinations such as 8‐oxodeoxyguanosine (8‐oxodGuo) and formylamine, and they pose significant challenges to DNA replication and repair fidelity [90]. Another type of tandem lesion occurs when ROS cause damage to two neighboring bases, commonly thymidine glycol (Tg) and 8‐oxo‐7,8‐dihydro‐2 ^′^‐deoxyguanosine (8‐oxodG) [91]. The formation of oxidative DNA lesions involving guanine and thymine is driven by the electrophilic nature of the guanine radical cation, which is highly susceptible to nucleophilic attack at its C8 position. This reactivity leads to the generation of two major guanine oxidation products: 8‐oxo‐7,8‐dihydroguanine (8‐oxoGua) and 2,6‐diamino‐4‐hydroxy‐5‐formamidopyrimidine (FapyGua) [92]. A notable nucleophilic interaction occurs when the N3 atom of thymine adds to the C8 of the guanine radical cation. This reaction results in the formation of intra‐strand cross‐links between guanine and thymine bases, which can occur in both single‐ and double‐stranded DNA. Following enzymatic digestion, these cross‐linked bases may be found adjacent to each other or separated by a 2 ^′^‐deoxycytidine unit, producing distinct lesions such as d(GpT) and d(G‐T) [93]. These cross‐links represent complex forms of DNA damage that can interfere with replication and repair processes, contributing to genomic instability.

#### 6.2.7. DPCs

DPCs represent a complex form of DNA damage involving covalent bonding between DNA and various cellular proteins, that block replication and transcription and play a role in aging, cancer, cardiovascular disease, and neurodegenerative disorders [94]. A broad spectrum of proteins—including GDP/GTP‐binding proteins, actin, histone H2B regulatory proteins, and structural proteins—has been implicated in DPC formation. One notable example is the thymine–tyrosine (Thy‐Tyr) cross‐link, which arises from the oxidation of tyrosine followed by the addition of a 5‐(uracilyl) methyl radical, forming 3‐[1,3‐dihydro‐2,4‐dioxopyrimidine‐5‐yl)‐methyl]‐L‐tyrosine. Additionally, amino acids such as serine and arginine, but not tyrosine, can act as nucleophiles and react with the guanine radical cation (G^+^) to form guanine‐based cross‐links [95]. In the context of interstrand cross‐links (ICLs), a critical step involves the nucleophilic addition of the cytosine 4‐amino group to a photo‐induced guanine radical cation, establishing a covalent bond between strands and severely impeding DNA replication and repair processes [96].

#### 6.2.8. Bypass of Oxidative DNA Lesions

To counteract mutations induced by DNA damage, all organisms are equipped with DDR mechanisms that accurately detect and repair genomic lesions [97]. These coordinated pathways ensure the faithful transmission of genetic material to daughter cells. However, when DNA damage persists unrepaired, it can traverse various cell cycle phases—S, G2, and M—potentially obstructing replication fork progression and precipitating RS. RS is a physiological condition marked by slowed or stalled fork movement, arising from factors such as DNA lesions, nucleotide depletion, or the trapping of replication/repair proteins, and is closely linked to genomic instability, with significant consequences for cell viability and disease development [98].

When the DDR proves insufficient, cells engage DNA damage tolerance (DDT) pathways to circumvent lesions and reinitiate DNA replication, thereby averting fork collapse and the generation of DSBs [99, 100]. The primary error‐free DDT mechanism is template switching (TS), which relies on HR machinery to utilize the newly synthesized sister chromatid as a repair template. Should cells fail to resolve replication impediments through error‐free means, alternative DDT strategies come into play, including repriming and replication restart past lesions, error‐prone lesion bypass, and post‐replicative gap filling—processes predominantly mediated by translesion synthesis (TLS) [101, 102] (Figure 2). While TS employs the nascent sister chromatid for accurate bypass, TLS utilizes specialized DNA polymerases to replicate across lesions, operating in either an error‐free or mutagenic manner [99, 103–105].

TLS is executed by a diverse array of polymerases, including Y‐family members such as Pol *η*, Pol *ι*, Pol *κ*, and REV1; A‐family Pol *θ*; B‐family Pol *ζ*; and X‐family polymerases Pol *μ* and Pol *λ* [106]. In contrast to high‐fidelity replicative polymerases (Pol *α*, *δ*, and *ε*), these TLS polymerases lack intrinsic 5 ^′^ → 3 ^′^ proofreading activity and possess larger, more accommodating catalytic pockets, enabling them to synthesize DNA across bulky, helix‐distorting lesions [107–109]. This structural adaptability prevents prolonged replication fork stalling and mitigates the accumulation of single‐stranded DNA (ssDNA) gaps. The outcome of TLS depends on nucleotide incorporation fidelity: correct base pairing yields error‐free bypass, whereas misincorporation introduces mutations [110, 111]. Given their propensity for mutagenesis, TLS polymerases are considered potential contributors to carcinogenesis [112].

Historically, TLS was conceptualized primarily as a mechanism for bypassing bulky DNA adducts “on the fly” during S‐phase [106, 113, 114], or alternatively, as a post‐replicative gap‐filling process that resolves ssDNA discontinuities across all cell cycle stages [115]. The “on‐the‐fly” mode entails a polymerase switch from replicative to TLS polymerases, a process mechanistically supported by PCNA ubiquitination (PCNA‐Ub), catalyzed by the RAD6‐RAD18 E2/E3 ubiquitin ligase complex [116–118].

### 6.3. The Base Excision Repair (BER) Pathway

Oxidative damage to DNA generates a wide range of lesions, each with distinct biological consequences. The 8‐oxodG lesion is not highly toxic to cells, but it is mutagenic, primarily causing G → T transversions because DNA polymerases efficiently incorporate adenine opposite the damaged guanine during replication—a process known as the GO pathway. Many replicative and repair polymerases, including *δ*, *κ*, *β*, *λ*, and *γ*, insert both dAMP and dCMP opposite 8‐oxodG. This lesion is also prone to further oxidation, producing more mutagenic derivatives such as guanidinohydantoin and spiroiminodihydantoin [119, 120].

Oxidation of 2 ^′^‐deoxyadenosine primarily yields 8‐oxo‐7,8‐dihydro‐2 ^′^‐deoxyadenosine (8‐oxodA) and 4,6‐diamino‐5‐formamidopyrimidine (FapydA). FapydA, the most abundant adenine lesion from gamma radiation, is found in both normal and cancerous tissues. Both lesions are only weakly mutagenic, but tandem 8‐oxodA lesions, induced by hydroxyl radicals, can block BER [121].

A major oxidized form of 2 ^′^‐deoxycytidine is 5‐hydroxy‐2 ^′^‐deoxycytidine (OH5dC), which arises spontaneously or from ROS exposure and can also form via dehydration of cytidine glycol. OH5dC can further deaminate to 5‐hydroxy‐2 ^′^‐deoxyuridine (OH5dU). Although OH5dC is readily incorporated into DNA, its replication is error‐prone, leading to C → T transitions.

Among thymine oxidation products, thymine glycol (Tg) is well studied. It blocks replicative DNA polymerases but is bypassed by translesion polymerases such as *η*, *κ*, *ν*, *β*, and *λ* with minimal mutation increases. Other oxidized thymine derivatives include 5,6‐dihydro‐thymine (dHT) and 5‐hydroxy‐5,6‐dihydro‐thymine (Th5).

5‐Hydroxymethyl‐cytosine (hmdC), an oxidized form of 5‐methylcytosine (5dmC), has been identified in mouse and human cells, particularly in Purkinje neurons and embryonic stem cells, where it is generated by TET1‐mediated hydroxylation [122]. Although human cells may accumulate about 20 hmdC lesions per cell daily, the glycosylase responsible for its removal remains unknown. However, deamination to 5‐hydroxymethyl‐uracil (hmdU) enables clearance via BER enzymes such as SMUG1, TDG, and MBD4 [123].

Beyond direct ROS‐induced DNA damage, chronic oxidative stress elevates LPO, producing reactive aldehydes like malondialdehyde, 4‐hydroxy‐2‐nonenal (HNE), and 2,4‐decadienal (DDE). These compounds react with DNA to form etheno‐base adducts, including 1,N^6^‐ethenoadenine, 3,N^4^‐ethenocytosine, N^2,^3‐ethenoguanine, and 1,N^2^‐ethenoguanine. These lesions are substrates for BER glycosylases. Notably, 1,N^2^‐ethenoguanine is repaired by BER and is highly mutagenic if left unrepaired; 1,N^6^‐ethenoadenine is preferentially excised by methylpurine DNA glycosylase (MPG or AAG); and 3,N^4^‐ethenocytosine is recognized but not excised by MPG, effectively sequestering the repair protein [124].

The BER pathway appears to have evolved specifically to counteract oxidative DNA damage. Most of the more than 20 proteins in the BER machinery participate in repairing one or more of these oxidative lesions. The following sections will detail the roles of individual BER proteins and their specificity in processing oxidatively damaged DNA [125].

## 7. The Effect of Free Radicals on Apoptosis

**Figure 5:**
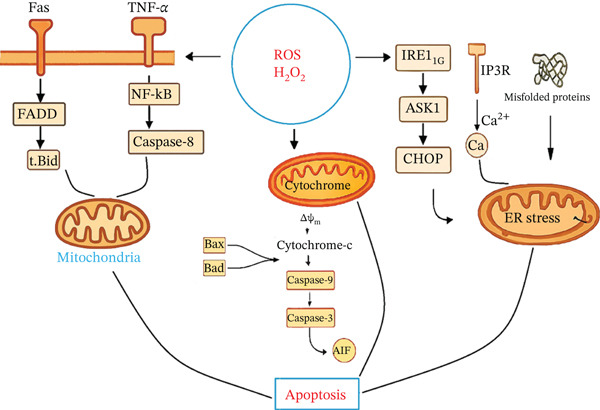
ROS‐mediated apoptosis: Integration of mitochondrial, death receptor, and ER–stress pathways.

ROS such as H₂O₂ exert dose‐dependent effects on cellular fate. At lower concentrations, ROS activate survival pathways and stimulate the tumor suppressor protein p53, which plays a pivotal role in maintaining genomic integrity. In contrast, higher doses of ROS trigger cell death mechanisms, notably apoptosis [126]. ROS particularly H₂O₂, affect apoptosis through three interconnected pathways: the intrinsic mitochondrial route, the extrinsic death receptor cascade, and the ER stress response (Figure 5). Under normal conditions, p53 is maintained at low levels due to ubiquitination by Mdm2, which targets it for proteasomal degradation. However, in response to cellular stress or DNA damage, p53 dissociates from Mdm2 and undergoes post‐translational modifications that stabilize it and prevent degradation, allowing it to bind DNA and exert its regulatory functions [127].

When stress levels are moderate, p53 promotes cell cycle arrest, DNA repair, and cellular senescence. It transcriptionally regulates apoptosis by repressing anti‐apoptotic proteins such as Bcl‐2, Bcl‐XL, IAPs, and survivin, while upregulating pro‐apoptotic factors [128]. In the nucleus, p53 activates genes like Fas, FasL, DR‐4, and DR‐5, as well as intrinsic pathway mediators including Bax, Bid, Puma, Noxa, and Apaf‐1, which initiate programmed cell death [127]. Beyond its nuclear role, cytosolic p53 translocates to mitochondria, where it directly interacts with pro‐apoptotic proteins Bak and Bax, inducing their structural reorganization and promoting mitochondrial outer membrane permeabilization (MOMP). This event facilitates the release of apoptogenic factors and drives apoptosis [129]. p53 also binds to anti‐apoptotic proteins such as Bcl‐2 and Bcl‐XL, further enhancing mitochondrial membrane permeabilization [130]. In HeLa cells, H₂O₂‐induced apoptosis is closely linked to p53 activity, particularly its role in Puma overexpression and Bax translocation to mitochondria [131].

### 7.1. ROS and the Mitochondrial Pathway

Mitochondria are the primary source of ROS in cells, largely due to electron leakage from the respiratory electron transport chain [132]. When mitochondrial DNA (mtDNA) is damaged, transcription of mitochondrial RNA (mtRNA) by proteins essential for the electron transport chain is impaired. This disruption compromises respiratory efficiency, increases ROS production, lowers mitochondrial membrane potential (*ΔΨ*m), and reduces ATP synthesis [133].

H₂O₂ plays a dual role in cell fate. It can trigger apoptosis via the mitochondrial pathway by releasing cytochrome c. Initially, H₂O₂ hyperpolarizes the mitochondrial membrane, but this is followed by a collapse in *ΔΨ*m, translocation of pro‐apoptotic proteins Bax and Bad into mitochondria, and subsequent cytochrome c release [134]. This initiates both caspase‐dependent and caspase‐independent apoptosis. In the caspase‐dependent pathway, cytochrome c activates caspase‐9, which in turn activates caspase‐3. In the caspase‐independent route, apoptosis‐inducing factor (AIF) is released from mitochondria and translocates to the nucleus to execute cell death [131].

Oxidative stress also activates the c‐Jun N‐terminal kinase (JNK) pathway, which phosphorylates and activates pro‐apoptotic proteins while inactivating anti‐apoptotic ones like Bcl‐2 and Bcl‐XL [135]. Additionally, mitochondrial calcium (Ca^2+^) levels influence cell death modality: high levels favor necrosis, while lower levels promote apoptosis. Under oxidative stress, both can trigger the opening of pores in the inner mitochondrial membrane (IMM), further exacerbating cell damage. ROS also accelerate Ca^2+^‐induced cellular degradation, reinforcing their central role in mitochondrial dysfunction and cell death [134].

### 7.2. ROS and the Death Receptor Pathway

ROS play a pivotal role in modulating extrinsic apoptosis pathways, particularly those initiated by death receptors such as TNF‐*α* and Fas. TNF‐*α* is a multifunctional cytokine that can either promote cell survival or trigger apoptosis depending on the cellular context and ROS levels [136]. At high concentrations, TNF‐*α* can induce necroptosis—a form of caspase‐independent cell death—through mechanisms involving ROS [137]. A positive feedback loop exists between TNF‐*α* and ROS, where each enhances the other’s production and signaling effects [138].

At low ROS levels, TNF‐R1 signaling activates NF‐*κ*B, which promotes cell survival by upregulating antioxidant enzymes like catalase and manganese superoxide dismutase (MnSOD). These antioxidants help neutralize mitochondrial ROS and prevent apoptosis. NF‐*κ*B also induces transcription of anti‐apoptotic genes such as IAP and Bcl‐XL, which inhibit caspase‐8 activation and block TNF‐R1‐mediated apoptosis [135, 138]. However, excessive TNF‐derived ROS can suppress NF‐*κ*B activity, thereby dismantling survival signaling and promoting cell death [139].

H₂O₂ also activates Fas‐mediated apoptosis. In HeLa cells, H₂O₂ upregulates Fas ligand (FasL) expression, leading to activation of FADD, caspase‐8, and caspase‐2. These caspases cleave Bid into t‐Bid, which translocates to mitochondria and amplifies intrinsic apoptosis by promoting MOMP and cytochrome c release [131]. Additionally, oxidative stress‐induced p53 activation serves as an upstream trigger for Fas‐mediated apoptosis, as observed in intestinal epithelial cells in mice. H₂O₂ also elevates mRNA levels of Fas and FasL, reinforcing the apoptotic signal [127].

ROS generation in cells is largely attributed to NOX family proteins, which are localized in subcellular compartments such as lipid rafts, caveolae, endosomes, and the nucleus [140, 141]. Upon ligand binding to Fas and TNF‐*α* receptors, lipid rafts form and recruit NOX enzymes, leading to ROS production. These ROS further activate death receptors and facilitate apoptosis. This intricate interplay between ROS, death receptors, and signaling pathways underscores the dual role of ROS in both promoting and regulating cell death.

### 7.3. ROS and the ER Pathway

Apoptosis and the unfolded protein response (UPR) are tightly regulated cellular outcomes in response to oxidative stress and protein misfolding. Under mild oxidative conditions, the UPR acts as a protective mechanism to restore homeostasis and maintain cell viability. However, when stress becomes severe or prolonged, apoptosis is triggered to eliminate damaged cells [142].

A key player in this transition is the ER stress sensor IRE1*α*, which activates apoptosis signal‐regulating kinase 1 (ASK1) and p38 MAPK, leading to the induction of the transcription factor CHOP and further ROS production [143, 144]. CHOP exacerbates oxidative stress by upregulating ERO1, an oxidase that generates ROS and enhances protein disulfide isomerase (PDI) activity. This promotes the formation of misfolded proteins and reoxidizes PDI, resulting in the accumulation of double disulfide bonds [145].

ERO1 also activates the inositol triphosphate receptor (IP3R), facilitating calcium (Ca^2+^) transfer from the ER to mitochondria, which can initiate mitochondrial‐mediated apoptosis [88]. ASK1 is further regulated by thioredoxin oxidation, enabling its interaction with Traf‐2 and promoting JNK activation—a critical mediator of ER stress‐induced apoptosis [132]. ROS themselves can directly trigger Ca^2+^ release from the ER lumen, intensifying mitochondrial stress [144].

Because of the close physical proximity of the ER and mitochondria via mitochondria‐associated membranes (MAMs), Ca^2+^ and other small molecules are efficiently exchanged between these organelles [146]. Elevated mitochondrial Ca^2+^ boosts metabolic activity by increasing NADH production for respiration and ATP synthesis. However, this also enhances ROS generation at complexes I and III of the electron transport chain [134]. Excessive mitochondrial Ca^2+^ activates the mitochondrial permeability transition pore (MPTP), releasing ATP, GSH, and cytochrome c—further amplifying ROS levels and apoptotic signaling [147].

During Bax‐dependent apoptosis, Bax‐Bak channels facilitate Ca^2+^ release from the ER to mitochondria, reinforcing inter‐organelle communication and apoptotic progression [128]. In HeLa cells, CHOP expression correlates with degradation of the calpain inhibitor calpastatin, exposing heightened activity of ER‐resident proteases such as calpain, caspase‐4, caspase‐12, and caspase‐7, which contribute to ER stress‐induced apoptosis [131].

### 7.4. Ferroptosis

Ferroptosis is a form of regulated cell death driven by iron‐dependent peroxidation of polyunsaturated phospholipids. Unlike other cell death pathways—such as apoptosis or necroptosis—ferroptosis is not executed through a particular protein effector; rather, it represents a membrane‐centered redox catastrophe in which chain‐propagating LPO outpaces cellular detoxification capacity [148, 149]. The glutathione peroxidase 4 (GPX4)–GSH system, which reduces phospholipid hydroperoxides, serves as the classical defense mechanism against ferroptosis. Failure of this system allows the accumulation of phospholipid hydroperoxides, which compromise membrane integrity and cellular viability [150, 151]. In addition to GPX4, other systems can intercept LPO in defined subcellular contexts, thereby raising the threshold for ferroptosis. For example, ferroptosis suppressor protein 1 (FSP1) regenerates reduced coenzyme Q (CoQ) and vitamin K—lipid‐soluble antioxidants that terminate the chain reaction of LPO—thereby protecting cells from ferroptosis, particularly under conditions of GPX4 insufficiency [152, 153]. The mitochondrial dihydroorotate dehydrogenase (DHODH)–CoQ and the GTP cyclohydrolase 1 (GCH1)–tetrahydrobiopterin (BH4) pathways also protect cells from ferroptosis through parallel mechanisms that inhibit LPO [154–156]. Conversely, oxidoreductases such as cytochrome P450 oxidoreductase (POR) and cytochrome b5 reductase 1 (CYB5R1), which promote LPO through their enzymatic activity, sensitize cells to ferroptosis [157–159]. Thus, rather than being a generic oxidative stress response, ferroptosis is better viewed as a failure to relieve lipid‐peroxidation pressure by various anti‐peroxidation defenses [160, 161].

Accumulating evidence supports a lipid‐centric framework for ferroptosis susceptibility. In addition to iron and oxidative stress—the essential drivers of ferroptosis—cellular vulnerability is often strongly shaped by the availability, distribution, and redox reactivity of peroxidizable membrane substrates, i.e., the balance between PUFA‐containing phospholipids and the regenerative capacity of anti‐peroxidation defenses. Here, we adopt this membrane‐centric perspective as a framework to emphasize how lipid composition interacts with iron and redox biology to set the threshold for ferroptosis [162].

## 8. The Effect of ROS on Telomere

ROS may directly damage DNA, shorten telomeres which are highly susceptible to ROS‐induced damage due to their high guanine content, inhibit telomerase, induce DDRs, and trigger senescence, all of which are significant contributors to the stabilization of cellular senescence [163]. Although numerous studies using genetically altered animal models targeting oxidative stress and mitochondrial function have produced contradictory findings regarding the relationship between oxidative damage and the ageing process, elevated ROS levels and mitochondrial dysfunction—characterized by inefficient metabolism—are widely recognized hallmarks of cellular senescence [164]. Senescence induced by replication, stress, and oncogenes is consistently associated with high ROS levels [165], and when DDR is activated by telomere uncapping or genotoxic stress, its downstream effectors further increase ROS production [166]. During senescence, ROS also act as signaling molecules; for instance, overexpression of activated RAS, BRAFV600E [167], p53, p21, and p16 has been shown to elevate ROS levels [164]. Several investigations have demonstrated that NOXs limit the replicative lifespan of human endothelial cells in vitro by generating ROS [168]. Moreover, ROS production and mitochondrial dysfunction—dependent on intact p53 and Rb tumor suppressor pathways—are closely linked to oncogene‐induced senescence. This dysfunction results in ATP depletion and AMPK activation, while mitochondrial ROS contribute to DNA oxidation [169]. Additionally, activation of pyruvate dehydrogenase in the TCA cycle, leading to increased respiration and ROS formation, has been shown to accelerate BRAFV600E‐induced senescence [167].

The tumor suppressor protein p53 plays a crucial role in mitochondrial regulation and cellular ageing. It has been established that p53 is involved in the transcriptional activation of genes related to mitochondrial apoptosis and its translocation to mitochondria enhances outer membrane permeabilization [164]. Moreover, p53 regulates the transcription of mitochondrial genes, thereby influencing mitochondrial function and accelerating ageing. In murine models, reduced expression of the Sco2 gene under p53 control led to impaired assembly of the COX II component encoded by mitochondrial DNA [164]. Activation of p53 has also been shown to inhibit the promoters of PGC‐1*α* and PGC‐1*β* genes in late‐generation mice with short telomeres, further implicating its role in mitochondrial dysfunction [170]. In both telomere‐dependent and ‐independent senescence, ROS production is diminished following RNA interference‐mediated knockdown of p53 and p21 [166]. Notably, DDRs in senescent cells create a persistent feedback loop that elevates ROS levels via p21 signaling. p21 also appears to be a key regulator of ROS through its modulation of DDR, MAPK, and TGF‐*β* stress‐induced signaling pathways [166]. Interestingly, ROS can influence DDR and promote senescence in a non‐autonomous manner, as senescent cells have been shown to induce DDR in neighboring cells through gap junction‐mediated intercellular communication [171].

Moreover, oxidation of telomeric DNA inhibits telomerase by the formation of 8‐oxoguanine (8‐oxoG) in G‐quadruplex DNA [172]. Results also confirmed the notion that telomerase is inhibited under oxidative stress conditions at chromosome ends in the absence of two antioxidant enzyme previously implicated in the protection of telomeres from oxidative damage, MTH1 and PRDX1 [173]. Indeed, increased telomere shortening in response to oxidative stress appears to be tissue‐dependent. However, most correlative studies (13 of 18) measured oxidative stress markers and telomere length in different tissue types—for example, oxidative stress in plasma and telomere length in DNA from blood cells. This approach likely precludes robust conclusions, as both telomere length and oxidative stress markers can vary across tissues [174, 175]. Similarly, measuring oxidative damage to lipids or proteins—rather than to DNA—is suboptimal for testing the effect of oxidative stress on telomere length, as damage levels to different biomolecules are not necessarily correlated [176, 177]. The timing of sampling is another critical parameter. Oxidative stress levels are likely to fluctuate much more rapidly than telomere length. Moreover, most effects of oxidative stress on telomere length are thought to manifest only after subsequent cellular replication, because single‐strand damage—more common than DSBs—shortens telomeres primarily during replication [178]. Therefore, the telomere‐shortening effects of an oxidative stress surge at a given time point may only become detectable later. This implies not only these experimental studies should measure telomere length sufficiently long after manipulation to allow for replication, but also that correlative studies should carefully consider sampling timing. One potential strategy would be to measure both “initial” telomere length and oxidative stress, measure “final” telomere length later (ideally accounting for cellular division timing in the target tissue), and then correlate telomere shortening with initial oxidative stress levels. Given that telomere length is largely determined by inheritance and early‐life conditions [179, 180], using the rate of telomere shortening rather than absolute length helps eliminate this “background noise” in correlations with oxidative stress. The life stage at which animals are sampled is also paramount. Telomerase is likely active during embryonic development and potentially at later life stages in particular tissues in some taxa [181]. This is important because it could mask the true relationship between oxidative stress and telomere shortening in vivo. Additionally, during growth periods, the end‐replication problem during cellular division is a key driver of telomere shortening, with consequences that researchers should consider. Rapid cellular division and the associated end‐replication problem during growth could reduce the likelihood of finding significant results in correlative studies by diminishing the relative proportion of telomere shortening attributable to oxidative stress. Conversely, rapid division could increase the likelihood of detecting significant results in experimental studies by rapidly converting single‐strand damage into actual telomere shortening. The nature of the experimental manipulation also warrants careful consideration. While antioxidant supplementation studies have been relatively successful in finding beneficial effects on telomere length, non‐significant results from such supplementation are unsurprising; supplementation is likely beneficial only when there is a genuine need for extra antioxidants, not when animals are not naturally resource‐limited [182]. This may explain why antioxidant supplementation has sometimes been beneficial only for specific subgroups of animals [183].

Other biases—including statistical and publication bias—may also skew our understanding of oxidative stress effects on telomeres. Bearing in mind that “correlation is not causation”, the absence of significant correlation should not be taken as evidence against causation. Type II error (false‐negative) must be carefully considered before drawing conclusions from non‐significant relationships, and sample sizes generally need to be very large to limit such errors. Publication bias favoring significant results is also likely to distort the overall picture in the literature—particularly in experimental studies focused on oxidative stress–telomere links, whereas correlative studies may be less susceptible to this bias as they often report these correlations as part of broader biological investigations [184].

## 9. Open Questions, Emerging Technologies, and Translational Perspectives

### 9.1. Unresolved Questions in ROS Biology

#### 9.1.1. Spatiotemporal Dynamics

The spatial segregation of ROS activities enables functional plasticity, where the same protein can participate in redox signaling in one compartment but adopt non‐redox functions in another, while simultaneously limiting collateral oxidative damage. Effective cellular adaptation therefore depends on robust communication between organelles. Understanding how this subcellular heterogeneity translates into distinct damage patterns and signaling specificity remains a fundamental challenge [185].

#### 9.1.2. Lesion Specificity and Repair

Perhaps the most intriguing paradox in oxidative DNA damage biology is this: OGG1 knockout animals amass significant levels of 8‐oxoG across their genome, yet they live a normal lifespan, develop no spontaneous tumors, and are surprisingly protected against inflammation. This suggests that lesion accumulation alone is not deleterious—and forces us to ask: what determines the non‐random, region‐specific distribution of oxidative lesions in the genome? [186].

#### 9.1.3. Inter‐Organelle Crosstalk

At the heart of inter‐organellar communication are redoxosome enzymes, which harness ROS to transmit signals through the chemical modification of proteins and lipids. Remarkably, ER chaperones and oxidoreductases are integral components of the redoxosome, casting ER oxidative protein folding as both a primary ROS source and a master regulator of the tri‐organellar membrane contact sites (MCS) that constitute the redox triangle. At these MCS, localized ROS bursts could directly facilitate signal transmission, perhaps via specialized ROS transporters. Simultaneously, ROS exert profound control over Ca^2+^ homeostasis, as critical Ca^2+^‐handling molecules—including IP₃Rs, SERCA pumps, and MCU regulators—are exquisitely sensitive to redox changes [187].

### 9.2. Emerging Technologies

#### 9.2.1. Real‐Time Redox Sensors

Genetically encoded redox biosensors represent a class of powerful tools for interrogating cellular redox processes with high spatiotemporal resolution. These sensors function by transducing the presence of a specific redox‐active analyte into a measurable fluorescence signal. This review provides a comprehensive survey of fluorescence recording methodologies, a detailed classification of biosensors based on their target analytes, and a discussion of recent chemigenetic developments, including both fluorescent and emerging strategies.

The past decade has seen the introduction of imaging tools—ranging from small molecule probes to genetically encoded sensors—that have enabled, for the first time, the direct visualization and quantification of redox reactions in living cells. Genetically encoded fluorescent probes such as HyPer, rxYFP, and roGFPs have been successfully employed across a wide spectrum of models, from cell cultures to transgenic animals. The considerable body of data now available facilitates a rigorous evaluation of the strengths and limitations of these probes [188, 189].

#### 9.2.2. Single‐Cell Profiling

To capture the complexity of cellular redox regulation, we developed SN‐ROP, a single‐cell mass cytometry‐based platform that enables real‐time monitoring of redox‐related pathway dynamics during oxidative stress. SN‐ROP simultaneously quantifies ROS transporters, antioxidant enzymes, oxidative stress products, and downstream signaling effectors, offering an unprecedented view of the redox landscape at single‐cell resolution. Across diverse cell types and experimental conditions, SN‐ROP has uncovered striking heterogeneity in redox responses, including coordinated shifts in CD8^+^ T cells following antigen stimulation and inter‐individual variations in CAR‐T cell persistence. Moreover, SN‐ROP analysis has revealed how microenvironmental factors—such as hypoxia and T cell exhaustion—shape redox balance, and has identified distinct redox signatures in patients undergoing hemodialysis. Together, these results underscore the power of SN‐ROP to illuminate complex redox networks and their significance for immune cell biology and disease pathogenesis [190].

#### 9.2.3. High‐Resolution Mass Spectrometry

A targeted and highly sensitive UHPLC‐QqQ‐MS/MS method has been developed for comprehensive DNA adductome analysis. The method is designed to expand the coverage of targeted DNA adducts by incorporating an expanded set of DNA lesions, thereby facilitating a more thorough characterization of DNA damage. This was achieved through the compilation of a comprehensive collection of DNA adduct standards and corresponding internal standards, enabling the establishment of a rapid, sensitive, and robust analytical platform for the simultaneous detection and quantification of multiple DNA adducts in a single run [191].

#### 9.2.4. CRISPR‐Based Functional Screens

While the CRISPR‐Cas9 system has been extensively employed for high‐throughput genetic screening, the implementation of genome‐wide sgRNA libraries poses considerable technical challenges. To address this, it was constructed a custom sgRNA library that is substantially smaller in scale—approximately one order of magnitude—than genome‐wide alternatives, specifically tailored for the interrogation of RNA binding proteins (RBPs). It was demonstrated the efficacy of this reagent in a genetic screen designed to identify RBPs that modulate cellular sensitivity or resistance to paraquat‐induced oxidative stress. This screen revealed that CSDE1 and STRAP, which physically interact, confer sensitivity to oxidative stress, whereas Pumilio family members (PUM1 and PUM2) confer resistance. Additionally, CRISPR‐mediated targeting of eIF4E1 and eIF4A1 provided protection against high‐dose paraquat, whereas disruption of eIF4E2 resulted in diminished cellular fitness under the same conditions [192].

### 9.3. Translational Roadblocks and Therapeutic Challenges

Randomized clinical trials employing rigorous methodologies have failed to demonstrate benefits of antioxidant supplements, with many reporting neutral or adverse outcomes. Systematic reviews that carefully account for bias and random error confirm that these supplements do not reduce the risk of cancer, cardiovascular disease, or mortality. Of particular concern, beta‐carotene, vitamin A, and vitamin E have been linked to increased mortality. These conclusions are reinforced by recent observational studies and have informed current dietary guidelines, which do not endorse antioxidant supplementation for primary prevention. For well‐nourished individuals, antioxidant supplements appear to be ineffective and potentially harmful, reinforcing the recommendation that antioxidants should be obtained through dietary intake rather than pharmaceutical formulations [193].

## 10. Conclusion

ROS occupy a dual role in biological systems, functioning as indispensable signaling molecules while simultaneously posing a significant threat to cellular integrity. At physiological levels, ROS regulate essential processes including gene expression, proliferation, differentiation, immune defense, and redox‐sensitive signaling pathways. However, perturbations in metabolic status, environmental exposures, mitochondrial dysfunction, and aging disrupt this balance, shifting ROS from regulatory mediators to cytotoxic agents. Elevated ROS initiate LPO, protein carbonylation, and a broad spectrum of oxidative DNA lesions—including base modifications, strand breaks, tandem lesions, and DPCs—that collectively compromise genomic stability and cellular viability. The resulting oxidative modifications, particularly within mitochondrial and nuclear DNA, promote mutagenesis, impair energy metabolism, and activate intrinsic and extrinsic apoptotic pathways. These cumulative effects contribute to the onset and progression of numerous pathological conditions and underscore the central role of redox imbalance in disease biology. It can be argued that free radicals play different and contradictory roles depending on their concentration, thus allowing for a re‐evaluation of diseases associated with free radicals, including alternative aging mechanisms, apoptosis as a pathway for cell death dependent on ROS, and the complexities of applied antioxidant strategies. This raises unanswered questions and sheds light on new techniques (real‐time redox sensors, single‐cell profiling, high‐resolution mass spectrometry, CRISPR‐based functional screens) to open the door to new future research and promising therapies based on the logic of the relationship between therapeutic effect and free radical concentrations.

## Author Contributions

Alkhaddour Aziz: conceptualization, literature search and review; data collection; evidence synthesis; drafting of the manuscript.

Zam Wissam: study conception and design; supervision of the review process; validation of extracted data; critical revision and editing of the manuscript.

## Funding

This research was funded by the Russian Science Foundation (grant no. 24‐15‐00268).

## Disclosure

Both authors read and approved the final manuscript.

## Conflicts of Interest

The authors declare no conflicts of interest.

## Data Availability

The data that support the findings of this study are available from the corresponding author upon reasonable request.
